# Evaluation of factors affecting physical activity and physical function in women with primary sjogren syndrome

**DOI:** 10.1590/1806-9282.20260748

**Published:** 2026-10-30

**Authors:** Oguzhan Mete, Serap Cinar, Emre Tekgoz, Seda Yurumez Colak

**Affiliations:** 1 University of Health Sciences, Gulhane Faculty of Physiotherapy and Rehabilitation, Department of Physiotherapy and Rehabilitation – Ankara, Turkey.; 2 University of Health Sciences, Gulhane Training and Research Hospital, Department of Internal Medicine, Division of Rheumatology – Ankara, Turkey.

**Keywords:** Physical activity, Physical fitness, Sjögren's syndrome

## Abstract

**OBJECTIVE:**

This study aimed to investigate the factors affecting physical activity and physical function in primary Sjogren syndrome.

**METHODS:**

In total, 63 women with primary Sjogren syndrome were examined for physical activity using the International Physical Activity Questionnaire, identified barriers to activity via the Self-Perceived Barriers for Physical Activity Questionnaire, and assessed physical function with the 30-s sit-to-stand and grip strength tests. Disease activity was measured with the EULAR Sjögren's Syndrome Disease Activity Index, symptom severity with the EULAR Sjogren's Syndrome Patient Reported Index, and fatigue using the Multidimensional Fatigue Inventory. Anxiety and depression levels were evaluated with the Hospital Anxiety and Depression Scale, while health perception was assessed with the Health Perception Scale.

**RESULTS:**

A one-unit increase in the HPS_Center of control score was associated with a 23.1-point increase in the median International Physical Activity Questionnaire total score (95%CIs 0.55; 63.52). An increase in the Hospital Anxiety and Depression Scale-Anxiety, Hospital Anxiety and Depression Scale-Depression, and MFI_total scores is associated with a rise in the Self-Perceived Barriers for Physical Activity Questionnaire score (0.69 [0.42; 1.13]), (1.07 [0.58; 1.41]), and (0.25 [0.1; 0.3]), respectively. Conversely, grip strength was associated with EULAR Sjogren's Syndrome Patient Reported Index, Hospital Anxiety and Depression Scale-Depression, and MFI_Reduced Motivation (-0.44 [-0.86; -0.01]), (-0.26 [-0.41; -0.08]), and (-0.22 [-0.48; -0.08]), respectively. However, the HPS_Self-awareness score had a positive effect on the 30-s sit-to-stand (0.18 [0.11; 0.3]).

**CONCLUSION:**

In women with primary Sjogren syndrome, emotional symptoms and fatigue were negatively associated with physical activity and function. Better health perception was positively associated with both. Health education and management of emotional disorders and fatigue can improve physical activity and function in primary Sjogren syndrome.

## INTRODUCTION

Primary Sjögren's syndrome (pSS) is an autoimmune disorder characterized by chronic inflammation that primarily affects the exocrine tissues, particularly the salivary and lacrimal glands. The hallmark symptoms include dryness, fatigue, and joint pain^1^. The prevalence of pSS is approximately 0.05 to 0.01%, and it affects women more than men^2^.

The decline in physical activity and physical function is an important issue that needs to be managed in rheumatic diseases^3^. Previous studies have highlighted that patients with pSS exhibit decreased levels of physical activity^4^ and physical function^5,6^. Additionally, the increased barriers to physical activity in pSS patients represent a significant concern^7^. To design an effective physical activity intervention and maintain physical function, a thorough understanding of these factors within the target population is crucial. However, studies on physical activity in pSS are limited^7^; research indicates that various factors can influence physical performance. Zangirolami-Raimundo et al. found a connection between cognitive function and complex physical tasks in postmenopausal women, highlighting the impact of cognitive factors on functional capacity in clinical populations. Therefore, it is crucial to investigate emotional and perceptual factors in pSS, where fatigue and mood disturbances are prevalent^8^. To the authors’ knowledge, there are no comprehensive studies focusing on factors that affect physical function. Therefore, this study aims to investigate the factors impacting these aspects in women with pSS.

## METHODS

### Participants and study design

A cross-sectional study was conducted at the Rheumatology Outpatient Clinic of Gülhane Training and Research Hospital from November 2024 to May 2025, approved by the University Ethics Committee (Decision number: 2024-477, 08.10.2024). It included women aged 40 to 65 (middle-aged adults) with pSS based on ACR/EULAR criteria^9^. Exclusion criteria included orthopedic or neurological diseases, inflammatory rheumatic diseases, psychiatric illnesses, pregnancy, and history of malignancy.

### Assessments

#### Dependent variables

The physical activity level was assessed using the International Physical Activity Questionnaire (IPAQ). It includes questions about the duration of vigorous and moderate physical activities, as well as walking. For each type of activity, the Metabolic Equivalent (MET) coefficient is multiplied by the total minutes spent on that activity, calculating the overall activity level. The overall activity level was recorded^10^.

The physical activity barriers were evaluated with the Self-Perceived Barriers for Physical Activity Questionnaire (SPBPA). It is a 17-item scale and is divided into four categories: body image and physical-social anxiety, fatigue and laziness, obligations and lack of time, and environment and facilities. A high score indicates a greater perception of barriers to physical activity^11^.

Physical function levels were assessed using the 30-s sit-to-stand test (30 STS) and grip strength test. In the 30 STS, participants sat on a chair without armrests, 45 cm high, with a straight back, feet flat, and arms crossed. They transitioned from sitting to standing and back within 30 s, and the number of successful repetitions was recorded^12^. For the grip strength test, a hydraulic hand dynamometer (Jamar^®^ Hand Dynamometer, 200 lb capacity, USA) was used. Participants sat upright on a stool without back support, feet flat, and arms at their sides. The tested arm was positioned with the elbow at 90 degrees. They squeezed the dynamometer as hard as they could, and the value was recorded^13^.

#### Independent variables

The disease activity was evaluated with the EULAR Sjögren's Syndrome Disease Activity Index (ESSDAI). It assesses 12 organ-specific domains, each with defined clinical parameters. Each domain is evaluated based on 3 to 4 levels of activity, with the conditions for each level clearly outlined. A higher score indicates a higher level of disease activity^14^. The symptom severity was assessed with the EULAR Sjogren's Syndrome Patient Reported Index (ESSPRI), which consists of questions that assess the severity of perceived fatigue, pain, and dryness as reported by patients, using a scale from 0 (no symptom) to 10 (highest level symptom). The overall score is calculated by summing them, meaning that a higher score indicates higher symptom severity^15^.

Fatigue was assessed using the Multidimensional Fatigue Inventory (MFI), which consists of subscales measuring general fatigue, physical fatigue, mental fatigue, reduced activity, and motivation. Participants rated the questions on a scale of 1 to 5, with each subscale score ranging from 4 (representing the best level of fatigue) to 20 (indicating the worst level of fatigue)^16^.

Anxiety and depression were assessed with the Hospital Anxiety and Depression Scale (HAD). It consists of a total of 14 items, with odd-numbered items assessing anxiety (HAD-A) and even-numbered items evaluating depression (HAD-D) tendency. Higher scores indicate a greater tendency to emotional disorders^17^.

Health perception was evaluated using the Health Perception Scale (HPS). It consists of 15 items and 4 subdimensions: certainty, locus of control, self-awareness, and importance of health. A higher score represents a higher health perception^18^.

### Statistical analysis

Data analysis was performed using IBM SPSS Statistics 27.0 and the quantreg library in R. Continuous variable distributions were assessed through skewness, kurtosis, the Kolmogorov-Smirnov test, histograms, and detrended normal Q-Q plots. Normal distributions were reported as mean±standard deviation (X±SD), while non-normal distributions were reported as median and interquartile range (IQR). Ordinal variables were expressed as frequency (n) and percentage (%). Quantile regression analysis (median, τ=0.5) was applied to evaluate the relationships between continuous dependent variables and their potential predictors. Quantile regression was chosen because it does not require normally distributed outcome variables and focuses on estimating the conditional median instead of the conditional mean. Additionally, median quantile regression is generally less sensitive to extreme observations compared to traditional mean-based regression methods, making it particularly useful when dealing with skewed outcome distributions. The effects of independent variables on the median of the dependent variable were reported with regression coefficients and 95%CIs. A statistically significant value was considered to exist if the confidence interval did not include zero.

For analysis where the dependent variable was categorical, logistic regression analysis was used. For parameters with cutoff values, categorization was performed using cutoff values; for those without cutoff values, the median value was used as the basis. First, the effects of independent variables were evaluated using univariate logistic models, and then, the variables were found to be significant and were included in the multiple models to perform multiple logistic regression analysis. The analysis results were reported with odds ratio (OR), 95%CI, and p-value. In all analyses, the statistical significance level was accepted as p<0.05.

## RESULTS

Of the 86 evaluated participants, 25 were excluded for various reasons: age criteria (n=15), not being volunteers (n=5), additional rheumatic disease (n=1), history of malignancy (n=1), pregnancy (n=1), and missing data (n=2); 61 participants with pSS (age: 54.41±7.34 years, body mass index: 28.02±5.05) were included. The clinical features and physical activity profile of participants were provided in Table 1. Table 2 showed quantile regression results between dependent and independent variables, and Table 3 presented logistic regression results and OR for dependent variables.

**Table 1:** Clinical characteristics and physical activity profile of participants.

Clinical symptoms	Descriptive value
Diagnosis period (months) [M (IQR)]	48.0 (64.0)
C-reactive protein g/L [M (IQR)]	1.5 (3.1)
Erythrocyte sedimentation rate mm/h [M (IQR)]	16.0 (15.0)
Schirmer right (positive) [n (%)]	46 (75.4)
Schirmer left (positive) [n (%)]	42 (68.9)
Hydroxychloroquine use [n (%)]	61 (96.8)
Immunosuppressant use [n (%)]	31 (49.2)
Presence of pain symptoms [n (%)]	52.0 (85.0)
Presence of dry mouth [n (%)]	48 (78.7)
Presence of dry eyes [n (%)]	53 (86.9)
Presence of dry skin [n (%)]	22 (36.1)
Presence of genital dryness [n (%)]	13 (21.3)
Presence of arthralgia [n (%)]	23 (37.7)
IPAQ total score	693 (712)
Vigorous physical activity (MET-min/week)	0 (0)
Moderate physical activity (MET-min/week)	0 (0)
Walking (MET-min/week)	594 (611)
Sedentary activity (sitting time) (daily/MET-min/week)	450 (270)

M: median; IQR: interquartile range; n: number; IU: international unit; mg/L: milligrams/liter; mm/h: milliliters/hour; IPAQ: International Physical Activity Questionnaire; MET: Metabolic Equivalent of Task; min: minutes.

**Table 2:** The quantile regression results between dependent and independent variables.

Independent variables	Dependent variables			
	IPAQ	SPBPA	Grip strength	30 s STS
ESSPRI	9.88 (-15.71; 80.57)	0.32 (-0.28; 1.45)	-0.44 (-0.86; -0.01)*	-0.14 (-0.29; 0.28)
ESSDAİ	-4.13 (-34.94; 5.92)	-0.05 (-0.3; 0.26)	-0.03 (-0.44; 0.13)	0 (-0.21; 0.19)
HADS-A	0 (-20.5; 42.28)	0.69 (0.42; 1.13)*	-0.05 (-0.31; 0.21)	0.09 (-0.2; 0.18)
HADS-D	0 (-25.72; 32.25)	1.07 (0.58; 1.41)*	-0.26 (-0.41; -0.08)*	-0.13 (-0.28; 0.26)
HPS_Total	0 (-10.35; 21.54)	-0.34 (-0.37; -0.22)*	0.03 (-0.09; 0.19)	0.04 (-0.09; 0.09)
HPS_Center of control	23.1 (0.55; 63.52)*	-0.44 (-0.71; 0.15)	-0.18 (-0.32; 0.29)	0.08 (-0.16; 0.17)
HPS_Self-awareness	-32.75 (-84.97; 87.18)	-0.39 (-0.9; 0.11)	0.46 (0; 0.88)	0.18 (0.11; 0.3)*
HPS_Certainly	0 (-19.93; 32.79)	-0.68 (-0.96; -0.4)*	0.14 (-0.14; 0.46)	0 (-0.2; 0.21)
HPS_Importance of health	-44 (-68.42; 22.43)	-0.84 (-1.2; -0.19)*	0 (-0.33; 0.3)	0.14 (-0.74; 0.26)
MFI_Total	0 (-8.28; 6.37)	0.25 (0.1; 0.3)*	-0.05 (-0.15; 0)	-0.04 (-0.06; 0.03)
MFI_General Fatigue	16.42 (-17.32; 44.56)	0.72 (0.09; 0.93)*	-0.16 (-0.43; 0)	-0.11 (-0.27; 0.06)
MFI_Physical Fatigue	0 (-49.34; 25.88)	0.65 (0.01; 1.19)*	-0.23 (-0.45; 0.01)	-0.1 (-0.28; 0.14)
MFI_Reduced Activity	-6.45 (-45.19; 10.15)	0.28 (-0.04; 0.77)	-0.22 (-0.39; 0.13)	-0.06 (-0.16; 0.21)
MFI_Reduced Motivation	-5.5 (-56.59; 25.17)	1.02 (0.84; 1.57)*	-0.22 (-0.48; -0.08)*	-0.13 (-0.36; 0.17)
MFI_Mental Fatigue	-19.25 (-46.18; 31.22)	0.66 (0.06; 0.92)*	-0.04 (-0.46; 0.33)	-0.13 (-0.18; 0.24)

ESSPRI: EULAR Sjögren Syndrome Patient Reported Assessment Index; ESSDAI: EULAR Sjögren Syndrome Disease Activity Index; HADS-A: Hospital Anxiety and Depression Scale-Anxiety; HADS-A: Hospital Anxiety and Depression Scale-Depression; HADS-D: Hospital Anxiety and Depression Scale-Depression; HPS: Health Perception Scale; MFI: Multidimensional Fatigue Index; IPAQ: International Physical Activity Questionnaire; SPBPA: Perceived Barriers to Participation in Physical Activity Scale; 30 s STS: 30 s sit-to-stand test. Quantile (median. τ=0.5) regression analysis. Significant results are highlighted in bold and marked with an asterisk (*).

**Table 3:** Logistic regression results and odds ratios for dependent variables.

Independent variables	Dependent variables			
	IPAQ	SPBPA	Grip strength	30 s STS
ESSPRI	p=0.163; OR 3.145	p=0.051; OR 4.00	p=0.123; OR 0.391	p=0.606; OR 0.735
ESSDAİ	p=0.373; OR 0.577	p=0.907; OR 0.946	p=0.907; OR 0.946	p=0.614; OR 0.786
HADS-A	p=0.383; OR 1.674	p=0.021; OR 3.619*	p=0.438; OR 0.667	p=0.933; OR 0.957
HADS-D	p=0.846; OR 0.844	p=0.022; OR 12.526*	p=0.438; OR 0.160	p=0.062; OR 0.128
HPS_Total	p=0.473; OR 1.528	p=0.043; OR 0.336*	p=0.097; OR 1.023	p=0.223; OR 1.900
HPS_Center of control	p=0.159; OR 2.269	p=0.433; OR 0.662	p=0.966; OR 0.662	p=0.933; OR 1.045
HPS_Self-awareness	p=0.929; OR 0.948	p=0.267; OR 0.545	p=0.433; OR 1.810	p=0.111; OR 2.444
HPS_Certainly	p=0.763; OR 0.840	p=0.071; OR 0.374	p=0.290; OR 0.871	p=0.666; OR 0.800
HPS_Importance of health	p=0.910; OR 0.938	p=0.037; OR 0.326*	p=0.791; OR 0.982	p=0.723; OR 0.833
MFI_Total	p=0.837; OR 1.125	p=0.004; OR 5.202*	p=0.973; OR 0.722	p=0.058; OR 0.367
MFI_General Fatigue	p=0.152; OR 2.400	p=0.071; OR 2.672	p=0.530; OR 0.667	p=0.034; OR 0.321*
MFI_Physical Fatigue	p=0.560; OR 0.710	p=0.072; OR 2.677	p=0.438; OR 0.579	p=0.022; OR 0.289*
MFI_Reduced Activity	p=0.688; OR 0.788	p=0.350; OR 1.643	p=0.299; OR 0.706	p=0.460; OR 0.677
MFI_Reduced Motivation	p=0.276; OR 0.532	p=0.026; OR 3.375*	p=0.515; OR 0.643	p=0.258; OR 0.556
MFI_Mental Fatigue	p=0.575; OR 0.718	p=0.219; OR 1.917	p=0.396; OR 1.100	p=0.645; OR 0.786

ESSPRI: EULAR Sjögren Syndrome Patient Reported Assessment Index; ESSDAI: EULAR Sjögren Syndrome Disease Activity Index; HADS-A: Hospital Anxiety and Depression Scale-Anxiety; HADS-D: Hospital Anxiety and Depression Scale-Depression; HPS: Health Perception Scale; MFI: Multidimensional Fatigue Index; IPAQ: International Physical Activity Questionnaire; SPBPA: Perceived Barriers to Participation in Physical Activity Scale; 30 s STS: 30 s sit-to-stand test; NS: not significant; OR: odds ratio. Logistic regression analysis and odds ratio. Significant results are highlighted in bold and marked with an asterisk (*).

Each 1-unit increase in the HPS_Center of control score is linked to an increase of 23.1 points in the median IPAQ total score (23.1 [0.55; 63.52]). Each 1-unit increase in the HADS-A and HADS-D scores is associated with increases of (0.69 [0.42; 1.13]) and (1.07 [0.58; 1.41]) in the median SPBPA score, respectively. Each 1-unit increase in MFI_Total score, MFI_General Fatigue, MFI_Physical Fatigue, MFI_Reduced Motivation, and MFI_Mental Fatigue scores is associated with increases of (0.25 [0.1; 0.3]), (0.72 [0.09; 0.93]), (0.65 [0.01; 1.19]), (1.02 [0.84; 1.57]) and (0.66 [0.06; 0.92]) points, respectively, in the median SPBPA score.

Each 1-unit increase in the ESSPRI score is associated with a decrease of 0.44 points in grip strength (-0.44 [-0.86; -0.01]). Similarly, increases in the HADS-D score and MFI_Reduced Motivation score led to decreases of approximately 0.26 points (-0.26 [-0.41; -0.08]) and 0.22 points (-0.22 [-0.48; -0.08]) in grip strength, respectively. Lastly, a 1-unit increase in the HPS_Self-awareness score is associated with an increase of about 0.18 points in the 30 STS score (0.18 [0.11; 0.3]) (Table 2).

Participants with high anxiety levels were 3.62 times more likely to have elevated SPBPA scores compared to those with lower anxiety levels (OR 3.619, p=0.021). Likewise, participants with high depression levels were 12.53 times more likely to have elevated SPBPA scores compared to those with lower depression levels (OR 12.526, p=0.022). Conversely, participants with high HPS scores are about 2.98 times less likely to score high on the SPBPA (p=0.043; OR 0.336). High scores on the HPS Importance of Health subscale also indicate a negative relationship with SPBPA, as those scoring high are 3.1 times less likely to have high SPBPA (OR 0.326, p=0.037). Additionally, individuals with high MFI_Total scores are 5.2 times more likely to have high SPBPA (p=0.004; OR 5.202), and those with high MFI_Reduced Motivation scores are approximately 3.3 times more likely to have high SPBPA (p=0.026; OR 3.375). In terms of the 30 STS, individuals with high MFI_General Fatigue are about 3.1 times less likely to score high compared to those with low general fatigue (p=0.034; OR 0.321). Similarly, high MFI_Physical Fatigue is associated with a 3.4 times decreased likelihood of high scores on the test (p=0.022; OR 0.289) (Table 3).

## DISCUSSION

The study explored factors influencing physical activity and function in pSS. It found that higher fatigue, anxiety, and depression significantly hindered physical activity, while symptom severity was negatively associated with physical function. Conversely, a better perception of health was linked to more physical activity and improved function. Notably, high anxiety and depression raised barriers to physical activity by 3.6 and 12.5 times, respectively, while high fatigue scores resulted in 3.4 times lower physical function.

The study identified fatigue, anxiety, and depression as negative factors that affect physical activity in pSS. The findings from limited studies support our results. Öztürk et al. found that higher fatigue, pain, anxiety, and depression levels in pSS patients created barriers to physical activity, with more severe symptoms negatively affecting activity levels^7^. Similarly, Ng et al. identified that increased depressive symptoms, fatigue, and daytime sleepiness were associated with reduced physical activity in pSS^4^. It was also on the link between emotional factors and physical performance align with findings from other female populations. Zangirolami-Raimundo et al.^8^ noted that cognitive function, closely tied to mood, correlated with physical performance in postmenopausal women. This suggests that the relationship between psychological well-being and physical function may be a broader issue affecting middle-aged and older women, not just those with pSS. Fatigue impacts physical activity in other rheumatic diseases. Katz et al.^19^ suggest that increased fatigue in rheumatoid arthritis may result from factors like physical inactivity, depression, obesity, and poor sleep. Their findings indicate potential interventions, such as promoting physical activity and addressing depression and obesity, to help reduce fatigue in RA patients.

The study also found that symptom severity, fatigue, and depression hinder physical functioning in pSS, aligning with findings from sarcopenia studies in this group. In their study on pre-sarcopenia and its effects in pSS, Öztürk et al. found that grip strength may be linked to depression and the severity of disease symptoms^20^. Additionally, Başaran and Büyükşireci examined the prevalence of sarcopenia in SjS and identified that the repeated sit-stand test, which measures physical function, is associated with disease activity, symptom severity, and increased fatigue^6^.

Fatigue is a prevalent and often debilitating symptom of pSS, significantly diminishing quality of life. Inflammation plays a key role in exacerbating fatigue for many patients, making chronic fatigue an unfortunate reality. This persistent exhaustion can lead to a cycle of behavioral disorders, where individuals may withdraw from physical activity, develop a fear of movement, and succumb to negative thought patterns about their condition. Negative factors contributing to chronic fatigue include emotional disorders like depression and anxiety, which can trigger each other. This creates a cycle of increased fatigue, worsening mood, avoidance of physical activity, and reduced physical capacity^21,22^. Unlike, health perception and awareness are important aspects of pSS. Research indicates that a positive health perception is associated with greater physical activity and improved physical function. While there are no specific studies on health perception in pSS, studies in other populations suggest that enhanced health perception and awareness foster a beneficial relationship with physical activity^23^. Breaking the cycle of fatigue, emotional distress, and physical inactivity may be achievable through structured exercise programs. Lino et al.^24^ found that a moderate exercise program for older people with type 2 diabetes mellitus significantly improved cognitive function, quality of life, and glycemic control. Although this group differs from those with pSS, it highlights how exercise can address multiple impairments like fatigue, emotional disturbances, and physical dysfunction simultaneously in pSS.

Our study highlights barriers to physical activity in patients with pSS. Enhancing physical activity and function is important an part of pSS management. Queiroz et al.^25^ provide direct evidence that physical activity interventions can improve clinically relevant outcomes in a population sharing characteristics with pSS who is under risk of sarcopenia^5,6^. Therefore, future studies should investigate whether tailored resistance exercise programs can enhance physical function and mitigate the metabolic effects of chronic inflammation and inactivity.

The first limitation of this study is the lack of analysis of medical treatments, comorbidities, and the duration of symptoms as potential factors affecting physical activity and function. Corticosteroids can impact mood, fatigue, and physical function, while immunosuppressants may have varying effects on the outcomes studied. Additionally, the duration of the disease and the presence of comorbidities can influence both physical activity and overall function. These factors may have significantly confounded the results; therefore, further studies should take them into account. Future research should also focus on the effects of treatment on these outcomes. Moreover, only middle-aged women were invited to the study, which meant that they were more likely to share similar physical profiles. However, this limitation affects the generalizability of our findings to the overall population of patients with pSS. Future research should include the elderly pSS population. Furthermore, physical activity was measured through self-reporting, so future studies could benefit from using measurement-based approaches, such as accelerometers. Lastly, the study had a relatively small sample size, especially considering the number of variables assessed. While the multivariable logistic regression analysis focused on six clinically relevant and predetermined main scale scores, the study also examined several subscales. As a result, the number of predictor-outcome associations analyzed was relatively high in comparison to the available sample size.

## CONCLUSION

In a clinical context, the reduction of fatigue and emotional distress could potentially lower the barriers to physical activity and enhance overall physical activity and function. Additionally, improving health perception and awareness may have beneficial effects on physical activity levels and functional fitness. The evidence from exercise intervention studies in older women^24,25^ suggests that structured physical activity programs could be effective in improving outcomes, supporting the need for clinical trials evaluating exercise interventions in pSS.

## Data Availability

The datasets generated and/or analyzed during the current study are available from the corresponding author upon reasonable request.
